# Evaluation of a 345 nm Femtosecond Laser for Corneal Surgery with Respect to Intraocular Radiation Hazard

**DOI:** 10.1371/journal.pone.0137638

**Published:** 2015-09-11

**Authors:** Johannes Menzel-Severing, Corinna Petsch, Theofilos Tourtas, Naresh Polisetti, Jörg Klenke, Katrin Skerl, Christian Wüllner, Christof Donitzky, Friedrich E. Kruse, Jan Kremers, Christian M. Hammer

**Affiliations:** 1 Department of Ophthalmology, University of Erlangen-Nuremberg, Erlangen, Germany; 2 WaveLight GmbH, Erlangen, Germany; 3 Department of Anatomy II, University of Erlangen-Nuremberg, Erlangen, Germany; Federal University of Rio de Janeiro, BRAZIL

## Abstract

**Purpose:**

We report our findings from a preclinical safety study designed to assess potential side effects of corneal ultraviolet femtosecond laser treatment on lens and retina.

**Methods:**

Refractive lenticules (-5 dpt) with a diameter of 6 mm were created in the right cornea of eight Dutch Belted rabbits. Radiant exposure was 0.5 J/cm² in two animals and 18 J/cm² in six animals. The presence of lens opacities was assessed prior to and up to six months following laser application using Scheimpflug images (Pentacam, Oculus) and backscatter analysis (Opacity Lensmeter 702, Interzeag). Ganzfeld flash and flicker electroretinogram (ERG) recordings were obtained from both eyes prior to and up to six weeks following laser application. At the study endpoint, retinas were examined by light microscopy.

**Results:**

Independent of energy dose applied, no cataract formation could be observed clinically or with either of the two objective methods used. No changes in ERG recordings over time and no difference between treated and untreated eye were detected. Histologically, retinal morphology was preserved and retinal pigment epithelium as well as photoreceptor inner and outer segments appeared undamaged. Quantitative digital image analysis did not reveal cell loss in inner or outer nuclear layers.

**Conclusions:**

Our analysis confirms theoretical considerations suggesting that ultraviolet femtosecond laser treatment of the cornea is safe for intraocular tissues. Transmitted light including stray light induces no photochemical effects in lens or retina at energy levels much higher than required for the clinical purpose. These conclusions cannot be applied to eyes with pre-existing retinal damage, as these may be more vulnerable to light.

## Introduction

Laser in situ keratomileusis (LASIK) has become a frequent corneal refractive procedure, but carries inherent risks [1]. These include epithelial ingrowth, dry eye and corneal ectasia as well as incomplete, free, irregular or small flaps, flap buttonholes and flap decentralization. With the introduction of femtosecond laser technology to corneal refractive surgery, some of these risks (i.e. those related to the use of a mechanical microkeratome) have been reduced. This is achieved by cutting the corneal flap using a femtosecond laser prior to stromal ablation by the excimer laser. To avoid the need for two different laser systems and to further increase the safety of refractive surgery, femtosecond laser techniques have evolved into “femtosecond lenticule extraction” (FLEx) and “small incision lenticule extraction” (SMILE). FLEx entails creation of a flap and a refractive lenticule that can be removed manually after opening the flap; all incisions are performed using the same femtosecond laser [2]. During SMILE, the lenticule is removed through one or two corneal incisions only a few millimetres in length, avoiding entirely the risk of flap-related complications [3–5]. The only available system for performing FLEx or SMILE currently is the VisuMax (Carl Zeiss Meditec AG, Jena, Germany), which uses an infrared laser source operating at a wavelength of 1043 nm.

The refractive laser examined here emits light at a wavelength of 345 nm, i.e. within the ultraviolet spectrum (UV-A). At shorter wavelengths, the threshold for tissue breakdown due to multi-photon absorption is lower than at longer wavelengths. Hence, laser energy levels required by a ultraviolet femtosecond laser to disrupt corneal tissue are tenfold lower than those required by an infrared laser [6]. In addition, light of shorter wavelength can be focused more precisely and may therefore allow more precise photodisruption of corneal stroma [7]. However, a large fraction of ultraviolet light is absorbed by the lens and may induce cataract formation [6]. For instance, acute cataract induction occurred within 24 hours following exposure to nanosecond laser pulses at a wavelength of 337 nm [8]. Ultraviolet light that is transmitted past the lens into the posterior segment of the eye can induce retinal damage [9]. In addition, previous studies determined that during flap cutting with the ultraviolet femtosecond laser, visible light reaches the retina, which has wavelengths centred around 440 nm [6]. Ham et al. had previously reported that the retina is particularly vulnerable to short-wavelength light within this spectral range, and showed photochemical damage to the rhesus retina following exposure to blue light (441 nm) [10]. This suggests that safety evaluations are required for ultraviolet refractive femtosecond lasers to exclude any potential damage to intraocular tissues.

Other safety evaluations have examined UV exposure thresholds and intraocular effects under different experimental conditions. For instance, Spoerl et al. report that homogenous irradiance with UV light at a wavelength of 370 nm and total radiant exposure of 5.4 J/cm^2^ (as is routinely used for corneal crosslinking therapy) is safe for intraocular tissues [11]. This is despite the fact that recommended exposure levels established by the ‘Guidelines on Limits of Exposure to Ultraviolet Radiation of Wavelengths Between 180 nm and 400 nm” [12] are surpassed, which supports the notion that data and safety recommendations from one medical application may not be useful for predicting side effects of another. Since ultraviolet refractive femtosecond lasers have not previously been assessed with regards to damage of intraocular tissues, we here report our findings from a preclinical safety study designed to assess potential side effects of refractive ultraviolet femtosecond laser treatment on lens and retina.

## Methods

### Experimental Animals

Eight female Dutch Belted rabbits weighing 2–2.5 kg were obtained from Kitayama Labes Co. Ltd. (Arai, Ina, Nagano Prefecture, Japan). Prior to the experiments, the animals were kept in colonies of a maximum of five animals with access to forage and water ad libitum. Experiments were conducted according to institutional guidelines and following approval of the experimental plan by the local government committee for the protection of animals (Regierungspräsidium von Mittelfranken, Ansbach, Germany; permit number 54–2532.1-16/11). For laser treatment and for lens- and ERG-measurements, the animals were anaesthetized with 50 mg/kg Ketamine (KetaVet, Pfizer Pharma GmbH, Berlin, Germany) and 10.5 mg/kg Xylazine (Rompun 2%, Bayer AG, Leverkusen, Germany). For additional local anesthesia of the cornea, oxybuprocaine hydrochloride eye drops (Conjuncain EDO; Bausch&Lomb, Berlin, Germany) were given. After surgical interventions the rabbits were kept in individual boxes with access to forage and water ad libitum. Animals were euthanized 6 months after surgery by intravenous injection of 300 mg pentobarbital (Narcoren; Merial GmbH, Sanofi, Paris, France) and the eyes were removed for histological analyses.

### Ultraviolet femtosecond laser treatment

**Fig 1:**
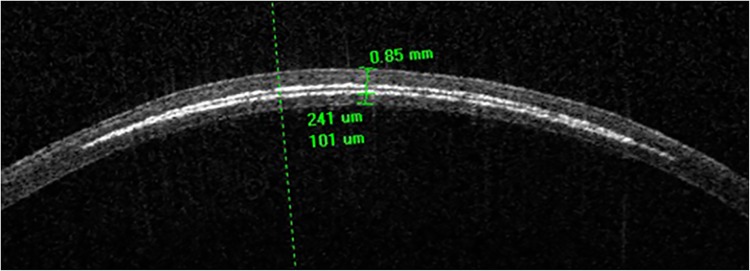
Anterior segment OCT scan of lamellar bed (white gas reflections) created by ultraviolet femtosecond laser.

A diode-pumped, solid-state, amplified femtosecond laser based on ytterbium technology was used to apply laser pulses (wavelength 345 nm, typical pulse duration <400 fs, repetition rate 1000 kHz) to the central cornea [13,14]. This created spatially confined photomechanical disruption of the corneal tissue, resulting in tangential intrastromal cleavage to create a refractive lenticule with a diameter of 6 mm and a theoretical correction of -5 dpt (the lenticules were not extracted; no extraction canals were created). Animals were treated with either 80 nJ per laser pulse and spot separation of 4 μm x 4 μm (“standard energy”, radiant exposure of 0.5 J/cm²) or 180 nJ per laser pulse and spot separation of 1 μm x 1 μm (maximum energy, radiant exposure of 18 J/cm^2^). During treatment, the eye was fixated by a custom-made suction ring to ensure correct and stable placement of the treatment zone. Correct depth of the posterior interface (100 μm from the endothelium) was monitored using optical coherence tomography (Cornea/Anterior Segment OCT CASIA SS-1000, Tomey, Erlangen, Germany; see Fig 1). In each animal the right eye was treated, while the left eye served as untreated control. Initially, two rabbits received standard energy while another two received maximum energy. Based on the results from this preliminary experiment, the remaining four animals all received laser treatment using maximum energy.

### Lens densitometry

**Fig 2:**
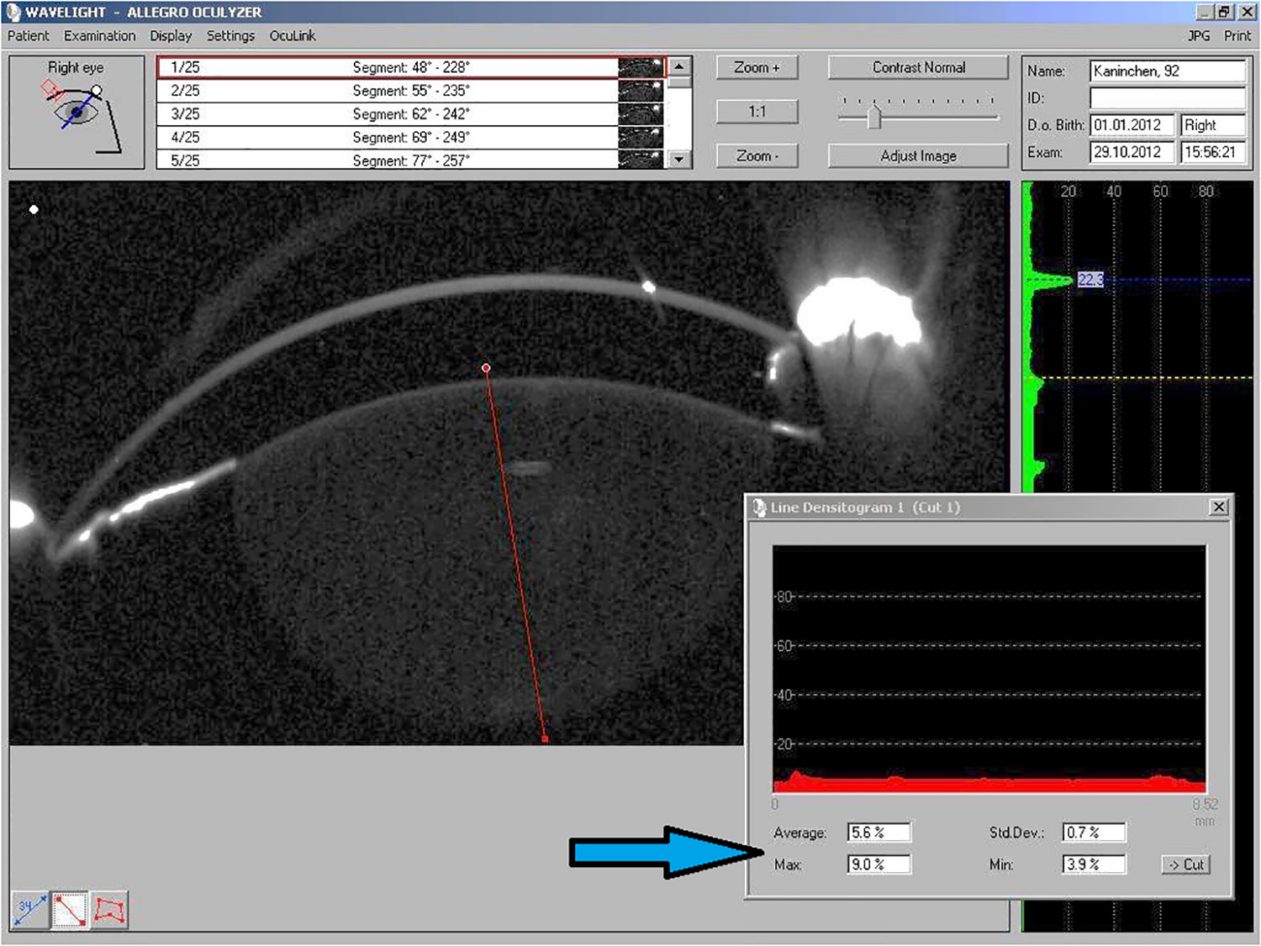
Scheimpflug image analysis of lens optical density. The same angle of measurement was used for all animals and at each time point. A line was drawn through the visual axis; mean and maximum densitometry values were recorded directly from the line densitogram (arrow).

To detect and quantify lens opacities, we used densitometry measurements from Scheimpflug images taken by the Pentacam system (Oculus Optikgeräte GmbH, Wetzlar, Germany), as well as back scatter assessments performed using the opacity lensmeter 702 (Interzeag AG, Schlieren-Zürich, Switzerland). Pupils were dilated with a drop of Tropicamide (Mydriaticum Stulln, 5.0 mg/ml, Pharma Stulln, Stulln, Germany) and of Phenylephrine hydrochloride (Neosynephrin POS 5%; Ursapharm, Saarbrücken, Germany). Measurements were obtained at predefined time points, i.e. one week and three days before laser treatment as well as two days, one week, two weeks, three weeks, four weeks, five weeks six weeks, two months, three months, four months, five months and six months after laser treatment. Data on mean and maximum optical density of the lens were extracted from Scheimpflug images as described in Fig 2. Two Pentacam measurements were obtained at each time point; these values were averaged for analysis. Five individual measurements were taken at each time point using the opacity lensmeter; again these values were averaged. Statistical analysis was performed using Microsoft Excel. A p-value below 0.05 was considered statistically significant.

### Electroretinography (ERG)

Baseline ERG measurements were performed 3 days before laser treatment. The measurements were repeated one day before laser treatment and in weekly intervals following laser treatment for a period of six weeks.

#### General setup

The animals were dark-adapted at least one hour prior to performing the measurements. All handling and preparations were performed under deep red illumination. Pupils were dilated with a drop of Tropicamide and of Phenylephrine hydrochloride in each eye. A contact lens electrode (Ocuscience, Henderson, USA) internally covered with Corneregel (Bausch & Lomb GmbH, Berlin, Germany) was placed on each cornea. Two needle electrodes, positioned subcutaneously in the ipsilateral temple, served as reference. The ground was a needle electrode placed subcutaneously at the tip of the ear. Impedance of active and reference electrodes were less than 10 kΩ. The animals were placed on a warming table and slid into a Ganzfeld bowl (Q450, Roland Consult, Brandenburg, Germany) that was used to deliver spatially homogeneous visual stimuli. The stimuli were delivered by white light emitting diodes. Stimulation and data recording were performed using the RetiPort system (Roland Consult, Brandenburg, Germany). The signals were filtered between 1 and 300 Hz. The following ERG measurements were performed:

### Scotopic flash ERGs

After five minutes of dark adaptation, the following sequence of scotopic flash ERGs were recorded: 0.0003 cd.s/m² flashes (n = 6–12) with 5 sec inter-flash intervals; 0.003 cd.s/m² flashes (n = 3) with 5 sec inter-flash intervals; after 20 sec of dark adaptation, 0.03 cd.s/m² flashes (n = 3) with 10 sec intervals; 60 sec of additional dark adaptation followed by 0.3 cd.s/m² flashes (n = 3) with 10 sec between flashes; finally after 120 sec of additional dark adaptation three flashes at 3 cd.s/m² were delivered with 14 sec between flashes. No reduction in response amplitude was observed after multiple flashes, indicating that there was no adaptation to the stimuli. In agreement with the ISCEV standards [15], the flash duration varied between 5μs and 5ms depending upon the required total energy.

#### Photopic flicker ERGs

Following the scotopic ERG measurements, the animals were light adapted to a 25 cd/m² background for at least three minutes. ERG recordings were performed while stimulating with trains of brief (5 ms or less) flashes upon the 25 cd/m² background. Temporal frequencies were 12, 18, 24 and 30 Hz and four flash intensities (0.095, 0.3, 0.95, 3.0 cd.s/m²) were used at each frequency. The mean luminance changed with stimulus strength and frequency. The mean luminance for 3 cd.s/m² flashes increased from 67 cd/m² at 12 Hz to 106 cd/m² at 30 Hz. Each measurement lasted 6 secs and commenced directly after stimulus onset. Stimulus onset artifacts were not observed. With these measurements cone driven flicker ERGs were obtained.

#### ERG data analysis

ERGs were analyzed using custom-written MATLAB programs (The MathWorks Inc., Natick, Massachusetts, USA) and Microsoft Excel spread sheets. Scotopic flash ERGs were filtered offline to remove contamination of the measurements by the oscillatory potentials (OPs) using a variable filter technique. This method has been described previously [16]. Briefly, the original recordings were Fourier transformed into the frequency domain where the OPs are defined by frequencies that are completely separated from those describing the conventional ERG signal. The minimal amplitude in the range between 50 and 100 Hz was determined. The amplitudes above this minimum described the OPs and were set to zero. The phases were not altered. The filtered signal was obtained by inverse Fourier transformation. The amplitude of the a-wave in the flash ERG was defined as the distance between the mean level before flash deliverance and the minimum of the a-wave. The amplitude of the b-wave was defined as the difference between the maximum of the b-wave and the minimum of the a-wave. Latencies of the a- and b-waves were also measured. Photopic flicker ERGs were Fourier analyzed and the amplitudes and phases of the fundamental (1st harmonic) and the 2nd harmonic components were extracted.

### Light microscopy

Samples were fixed for 24 hours at room temperature in a solution containing 2.5% glutaraldehyde in Sörensen’s phosphate buffer [0.1 M KH_2_PO_4_, 0.1 M Na_2_HPO_4_ × 2 H_2_O; pH = 7.2]. They were then post-fixed in 2% osmium tetroxide for 1.5 hours at room temperature and dehydrated in an ascending series of alcohols. After embedding the tissue in Epoxy resin, sagittal semithin sections of 1 μm thickness were cut with a microtome (Ultracut E, Reichert-Jung Inc, Buffalo, USA), stained with toluidine blue (retina) or hematoxylin and eosin (iris), coverslipped, and viewed with a Keyence microscope (Biorevo BZ-9000E, Keyence Corporation, Osaka, Japan). Quantitative analysis of cell nuclei in retinal sections was performed by counting nuclei from the outer and inner nuclear layers on digital images. For each experimental animal, one image was obtained at a magnification of 600x and all perikarya within one field of view were counted for each layer. Statistical analysis (t-test) was performed using Microsoft Excel.

## Results

Evaluation of preliminary data obtained from four initial animals suggested no difference between standard and maximum energy (two animals each; data not shown). Hence, the remaining four animals all received laser treatment using maximum energy, and overall statistical analysis was carried out using only data from animals that received maximum energy (n = 6).

### Clinical evaluation of the anterior segment

**Fig 3:**
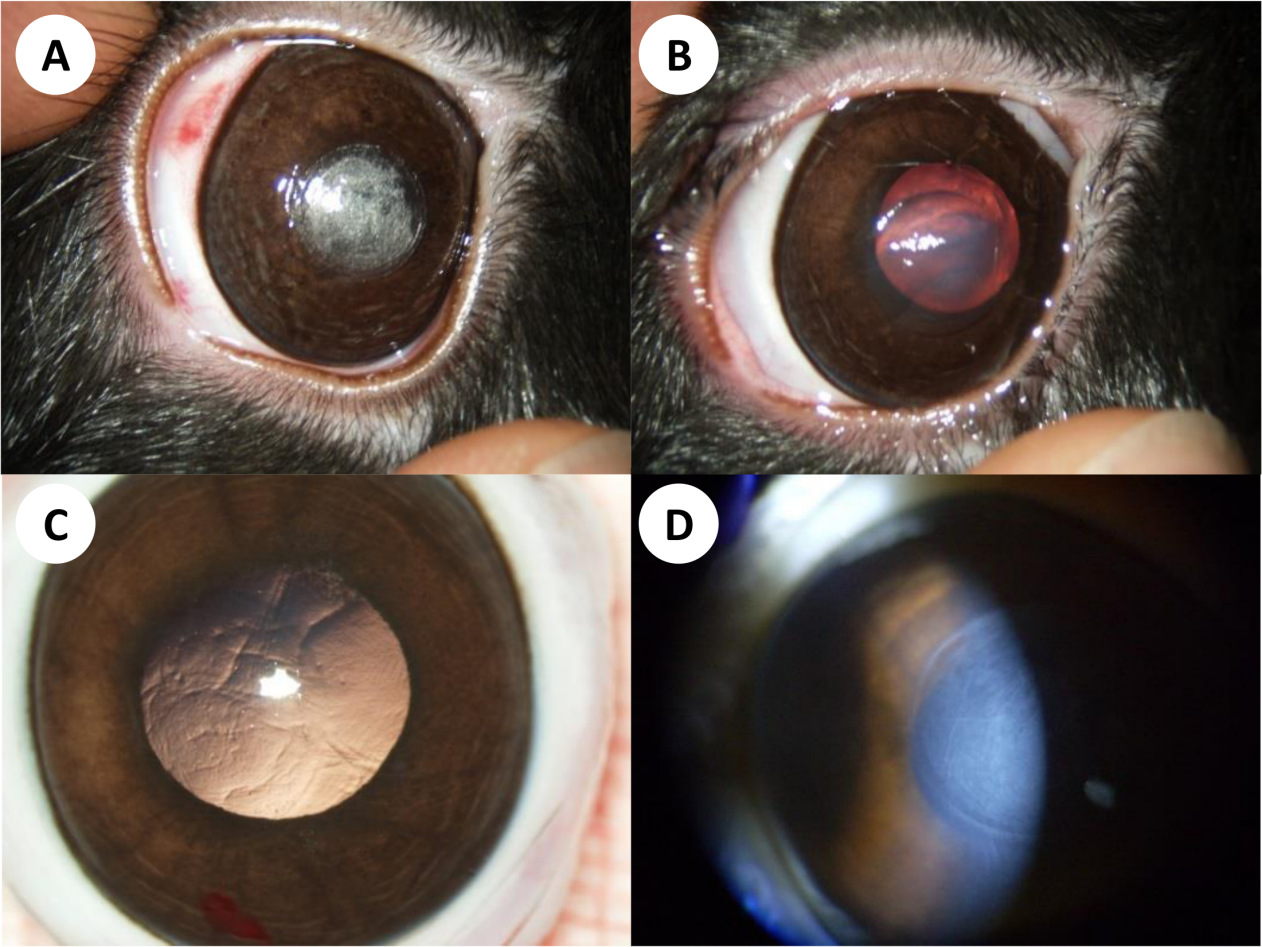
Representative anterior segment findings in one experimental animal (K89). (A) Gas bubbles immediately after UV-laser treatment with maximum energy indicate successful thermomechanical disruption of corneal stroma. Mild subconjunctival haemorrhage is caused by the suction ring used to fixate the eye during the procedure. (B) At postoperative day 7, localised corneal edema was seen in most eyes receiving maximum laser energy. (C, D) Six months after surgery, a smooth corneal surface and clear media were observed in all cases. Slit lamp examination reveals mild intrastromal haze at the site where the stromal cut was performed.

Macroscopic images of ocular findings following laser treatment are shown in Fig 3. Immediately after laser application small but abundant gas bubbles could be observed (Fig 3A). Following maximum laser energy application, well-circumscribed corneal edema developed (Fig 3B) which cleared within two weeks (Fig 3C). At the study endpoint (6 months), the anterior segments showed mild intrastromal haze within the cleavage plane but no other pathological findings (Fig 3D). Intraocular or ocular surface complications did not arise during the course of this study.

### Lens densitometry

**Fig 4:**
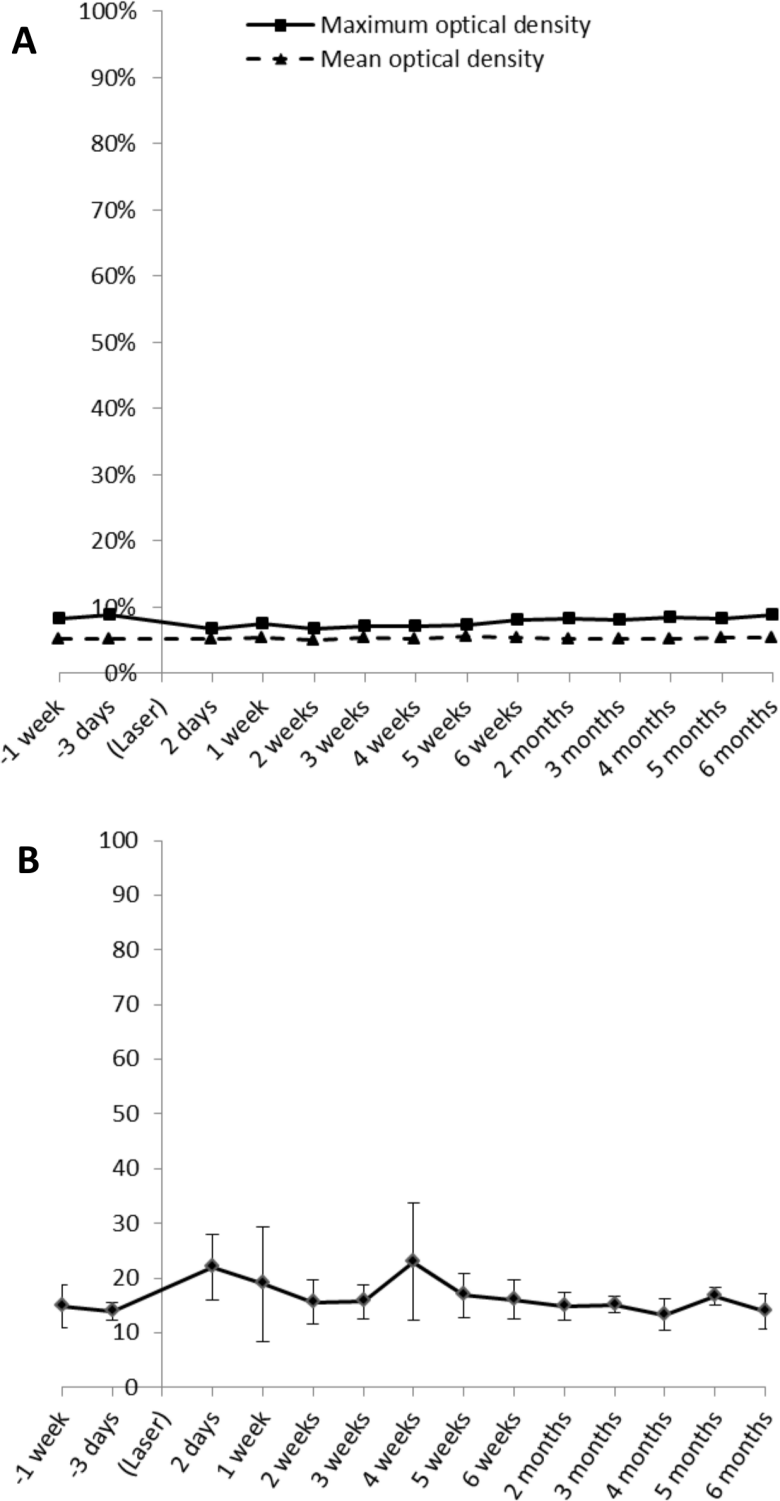
A: Pentacam densitometry values of experimental animals treated with maximum laser energy (Mean values; SD is too small to be seen in this diagram). No increase in optical density (i.e. no signs of cortical cataract formation) can be observed up to 6 months after intrastromal laser application. B: Opacity Lens Meter readings of experimental animals treated with maximum laser energy (Mean ± SD). No signs of nuclear cataract formation can be observed up to 6 months after intrastromal laser application.

Average lens density values (Pentacam) ranged below 5% at baseline and remained unchanged throughout the study (Fig 4A). Also, no focal opacities were seen, as judged by the analysis of peak density values. These data are in agreement with opacity lensmeter readings which, like Pentacam measurements, did not show any significant changes from baseline to postoperative month six (Fig 4B). Normal values for this instrument range between 4 and 25 in humans [17].

### ERG

**Fig 5:**
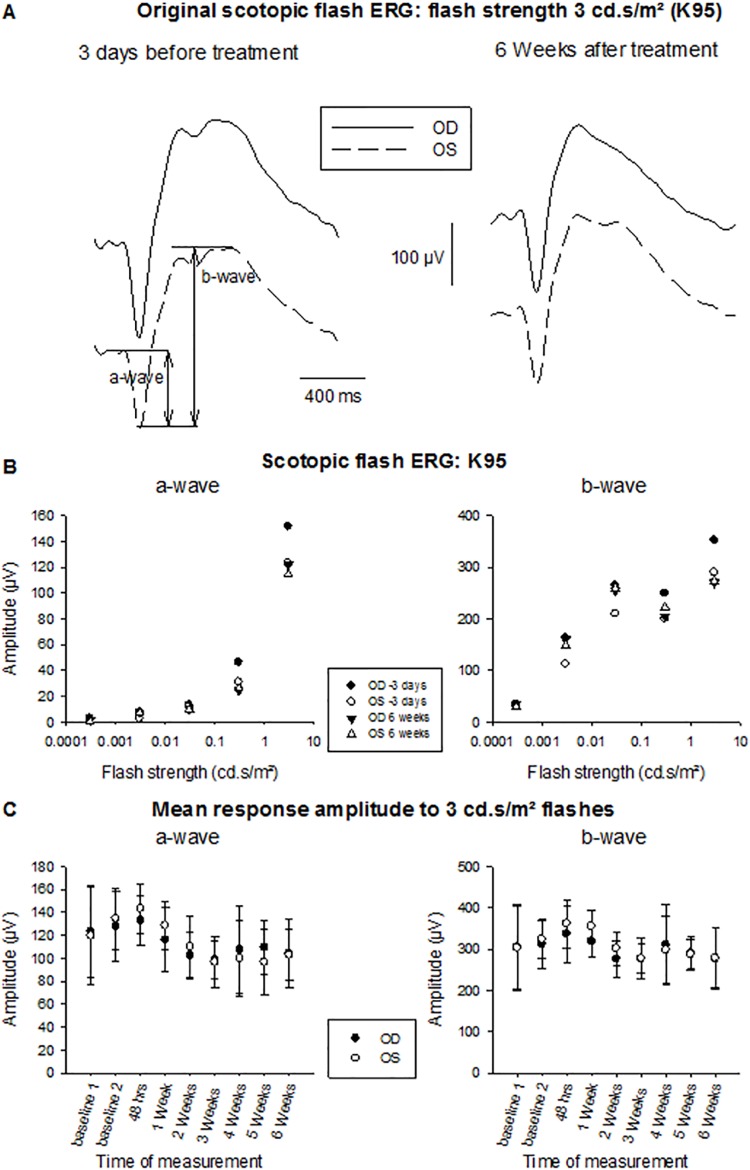
Scotopic flash ERGs. A: Original recordings in the treated (right) eye (drawn curves) and control (left) eye (dashed curves) of one animal (K95) before and 6 weeks after laser treatment. Flash strength was 3 cd.s/m². B: Amplitudes of the a- (left plot) and the b-wave (right plot) given as a function of flash strength. Recordings are from the same animal whose responses are shown in panel A, 3 days before treatment (circles) and 6 weeks after treatment (triangles). Closed symbols: right eye; Open symbol: left eye. C: Mean amplitudes of the a- (left plot) and b-wave (right plot) from the group receiving maximum laser energy (n = 6) obtained with 3 cd.s/m² flashes at different time points relative to the treatment.

Fig 5A shows filtered ERGs to 3 cd.s/m² flashes recorded in the left and right eyes of a rabbit (K95) whose right eye received a maximal radiant dose during treatment. The shown responses were measured three days before treatment (left responses) and six weeks after standard treatment (right responses). There are no differences between the responses recorded in the right (treated) and left (control) eye. In addition, no obvious changes in the responses in this time period can be observed. Fig 5B displays the amplitude of the a- and b-waves in the two eyes of the same animal 3 days before and 6 weeks after treatment given as a function of flash strength. Again there are neither ERG amplitude differences between left and right eyes nor between recordings before and after treatment. Fig 5C shows the mean a- and b-wave amplitudes, obtained with 3 cd.s/m² flashes, for those animals that received maximal laser dose (n = 6) as a function of time. No obvious changes in time can be observed and the amplitudes for the two eyes are very similar. The implicit times (delays) of the two components were also similar in the two eyes and stable in time. Very similar results were obtained for the two animals that received standard treatment doses (not shown).

**Fig 6:**
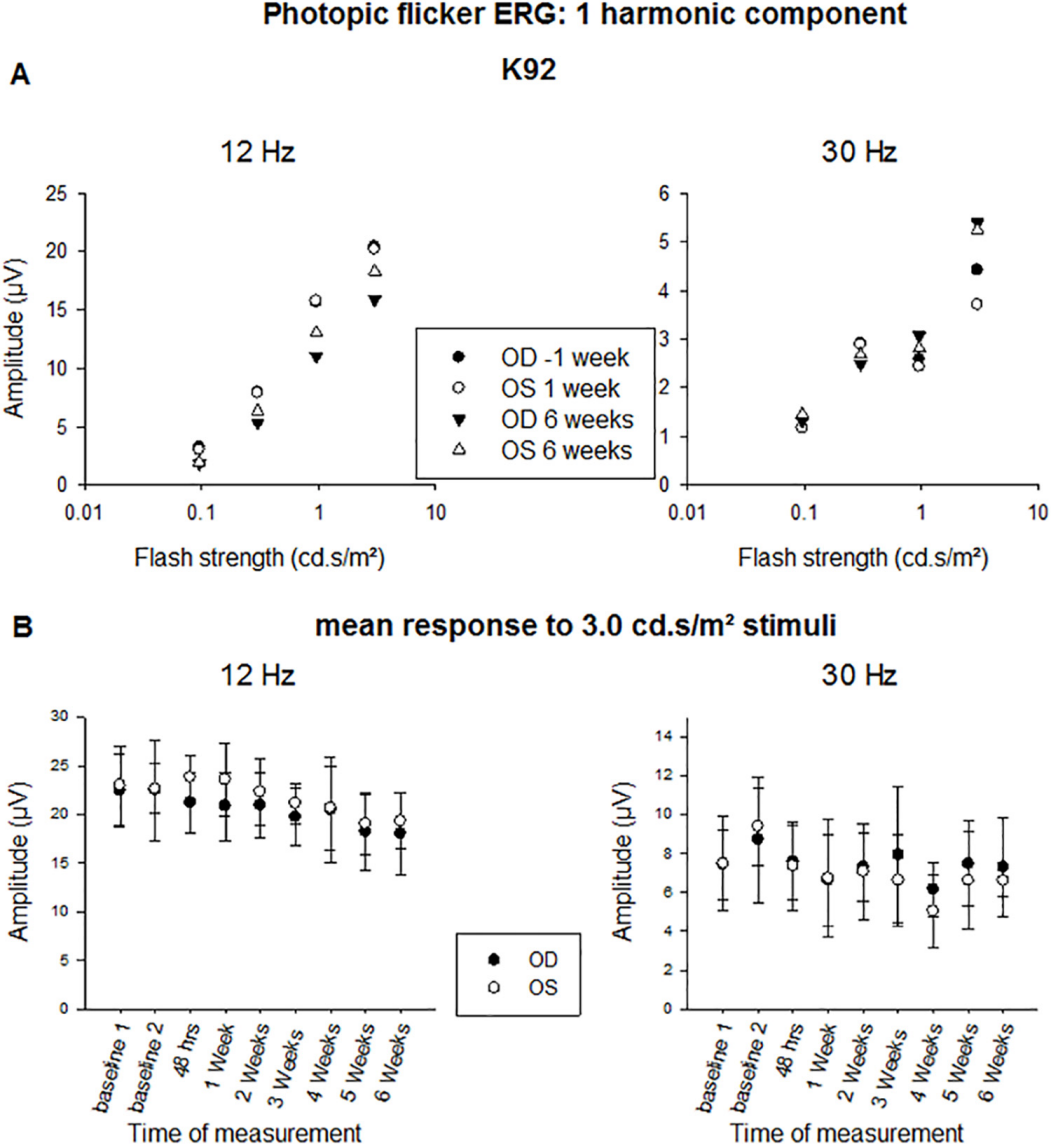
Photopic flicker ERGs. A: Amplitudes of the 1st harmonic component to 12 Hz (left plot) and 30 Hz (right plot) stimuli given as a function of flash strength. The recordings were performed 3 days before (circles) and 6 weeks after treatment (triangles). Closed symbols: right eye; Open symbol: left eye. B: Mean amplitudes of the 1st harmonic component to 12 Hz (left plot) and 30 Hz (right plot) stimuli obtained with 3 cd.s/m² flashes at different time points relative to the treatment. Panel A shows representative results from one animal (K92), while panel B shows mean results from the group receiving maximum laser energy (n = 6).

Fig 6A displays the amplitudes of the 1st harmonic (fundamental) component of the 12 Hz and 30 Hz photopic flicker ERGs as a function of flash strength obtained in another animal (K92) that received the maximum treatment dose in the right eye. In agreement with the results for the scotopic flash ERGs, the amplitudes were stable over time and did not differ between treated and control eye. Fig 6B displays the mean amplitude of the 1st harmonic component after stimulating with 3 cd.s/m² flashes measured in the six animals that received maximal radiant doses. The treatment had neither an effect on the amplitudes of 1st harmonic components of the responses at the two other temporal frequencies (18 and 24 Hz) nor did it have an effect on the 2nd harmonic components at all frequencies. Overall, no systematic effect was found on the phases of all components.

### Histology

**Fig 7:**
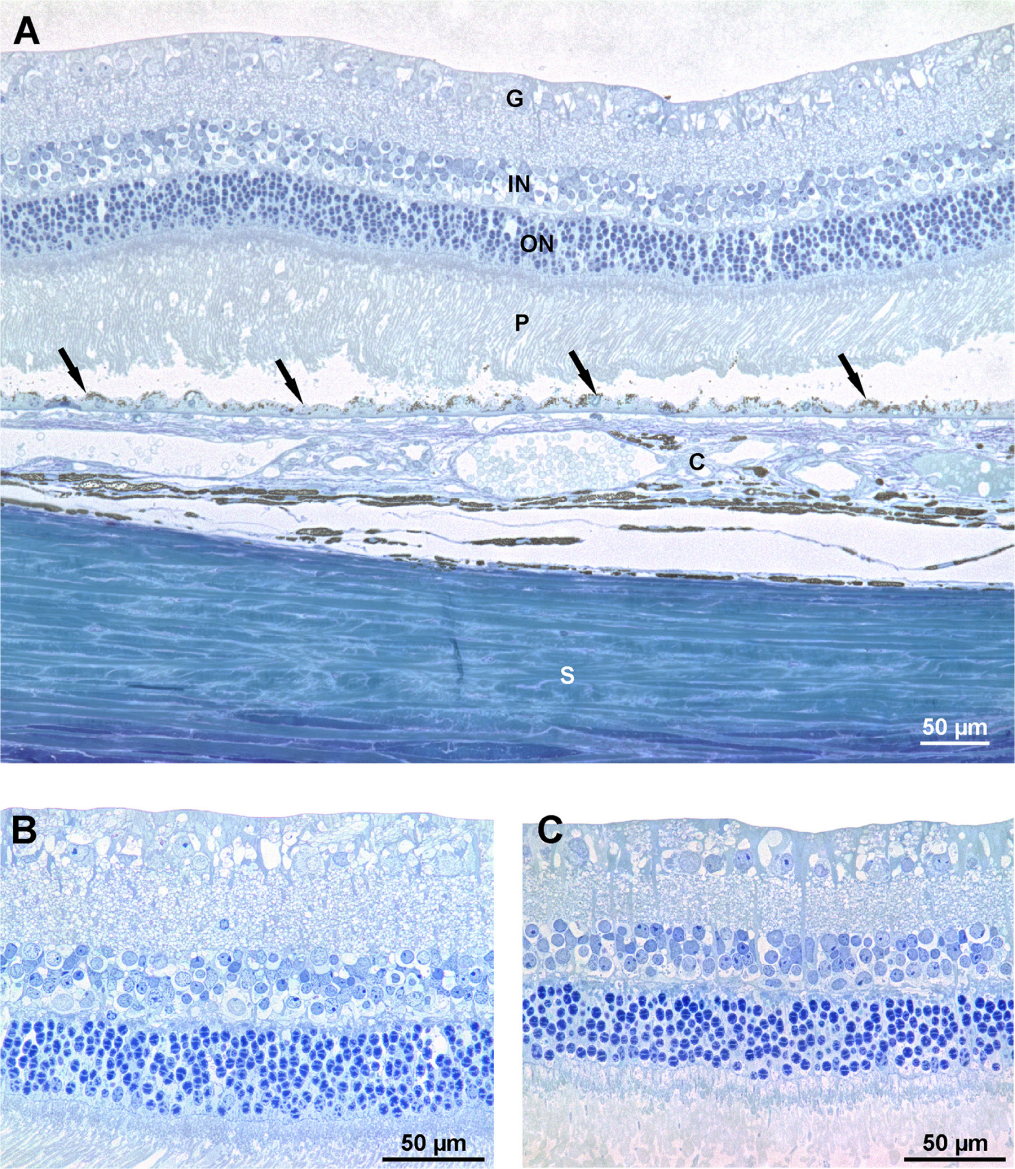
Representative retinal sections. **A:** General aspect of a rabbit retina following UV femtosecond laser treatment to the cornea with maximum energy. All retinal layers appear unaltered. (G: ganglion cell layer; IN: inner nuclear layer; ON: outer nuclear layer; P: photoreceptor outer segments; Arrows: retinal pigment epithelium; C: choroid; S: sclera.) **B:** Enlarged view of panel A. **C:** Control section from an untreated eye. Semithin sections stained with toluidine blue.

**Fig 8:**
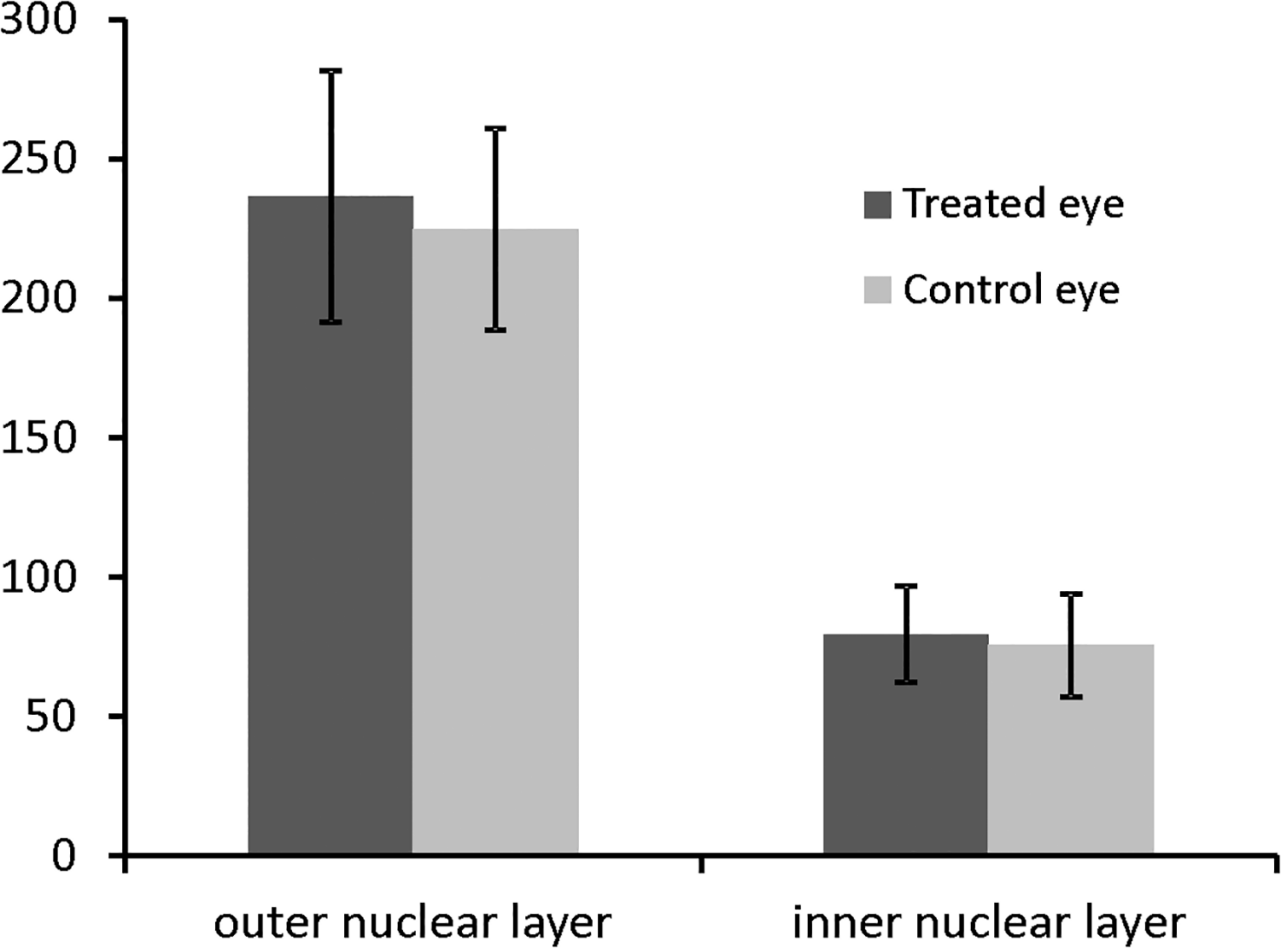
Quantitative analysis of cell nuclei in retinal sections. Nuclei from the outer and inner nuclear layers (the former containing the photoreceptor perikarya) were counted on images taken at a magnification of 600x (see Fig 7B/7C). Mean values (±SD) from six animals that received maximum laser energy are given. Differences between treated eyes and contralateral control eyes are not statistically significant (p>0.5).

Retinal sections showed no structural alterations in any of the retinal layers (Fig 7). This was further confirmed by quantitative image analysis, showing no significant change of nucleus counts in both the outer and inner nuclear layers (Fig 8). No alterations were found in iris sections of treated or untreated eyes (S1 Fig).

## Discussion

It is well established that UV radiation can be harmful to ocular structures [18]. More than two thirds of UV light applied to the cornea is transmitted through the tissue and can reach the intraocular structures, with similar transmittance values reported for human [19] and rabbit eyes [20]. While light of longer wavelengths primarily causes thermal injury, UV light causes predominantly photochemical damage, i.e. chemical changes in the irradiated molecules with resultant cell damage or death [21,22]. The International Commission on Non-Ionizing Radiation Protection defines a safety threshold of 13 J/cm^2^ for UV radiation for the entire eye at 345 nm [12]. However, these recommendations may not be useful in the special case where femtosecond UV laser pulses are used for intrastromal photodisruption. Therefore, dedicated analysis of intraocular effects during this application is required. Le Harzic et al. have examined the transmission of a collimated laser beam of 345 nm wavelength through cornea and lens and onto the retina [6]. Their data suggest that more than 50% is transmitted through the cornea, but less than 15% is transmitted beyond the lens, with only 5% reaching the retina. Under flap cutting conditions, these values are further reduced, and less than 2% of UV radiation reaches the retina [6].

To assess effects on corneal endothelium, we recently examined the impact of UV femtosecond laser treatment in rabbit and porcine corneas ex vivo [23]. Our data suggest that intrastromal lamellar cuts in the posterior corneal stroma can be performed without compromising corneal endothelial cell survival. This was the case even when the energy dose was set to 5.1 J/cm^2^, which is 10 times higher than the standard energy required for intrastromal cutting. In the present study, the energy dose was 36 times higher than the standard. It appears quite likely that under these conditions, damage to corneal endothelium did occur, given the significant stromal edema (Fig 3). However, such a high energy level is not necessary to achieve a stromal cut and would therefore never be used in a clinical setting. Here, we have chosen this energy level to rule out intraocular effects with an ample safety margin. Trost et al. recently reported good corneal tissue tolerability of a 355 nm nanosecond corneal refractive laser [24]. However, follow-up time did not exceed 24 hours and no intraocular tissues have been evaluated. Our results suggest that deleterious long-term effects on adjacent tissues, in particular lens and retina, do not occur even at laser energy levels so high that corneal damage is observed.

ERG measurements were discontinued after 6 weeks since it was deemed unlikely that retinal damage would become manifest weeks after UV irradiation. On the contrary, cataract development is known to be a long-term sequel of UV irradiation. Therefore, an extended follow-up of 6 months was chosen for lens opacity measurements. No biomicroscopic signs of lens changes were observed throughout the course of this study. To achieve a more objective assessment, it had been suggested that the Pentacam Scheimpflug system should be used to classify lens opacities [25,26] in longitudinal studies [27]. Other studies in rabbits have also used Scheimpflug imaging to assess cataract formation following femtosecond laser treatment to the lens [28]. Therefore, we have adapted the method described by Pei et al. [29] to obtain quantitative and objective measurements of lens density that are in good agreement with the Lens Opacities Classification System III. Using this approach, no cataract formation could be detected during a follow-up period of 6 months, which, together with the absence of changes measured with the opacity lensmeter, suggests safety of the intracorneal application of the UV femtosecond laser. However, Pei and colleagues measured predominantly nuclear cataracts. In addition, while opacity lensmeter readings were found to correlate well with nuclear lens opacities, they may be of limited value in measuring cortical opacities [30]. These considerations are of relevance in the context of our study, as it was suggested that acute UV exposure is related predominantly to the formation of cortical cataracts [31]. Ullrich et al. [32] have developed a method to objectively measure cortical opacities using the Pentacam system. Their approach relies on counting the number of Scheimpflug images (out of a series of 25) that show significant cortical cataract, with significant being defined as more than 20 density units. It is worthwhile to point out that in our series, at no point density values above 20 were recorded, thereby effectively ruling out the presence of cortical cataract.

It is believed that during environmental exposure to sunlight, UVB radiation is more phototoxic to the lens than UVA radiation [21]. Therefore, while UVA light may still contribute to progressive opacification of the lens, it may be beneficial with respect to lens clarity that the laser evaluated here emits light within the UVA band only. Also, it has been suggested that the incident angle of light rays determines their cataractogenous effect. Oblique rays that reach the equatorial region of the lens where its germinative center lies may have a stronger effect than axial rays that pass directly though the pupil [33]. Since for most applications the refractive laser is applied centrally over the cornea, much of the applied energy may be shielded by the iris.

Light that is transmitted to the posterior pole may provoke hazardous effects at the level of the retinal pigment epithelium (RPE) and the photoreceptors. Photoretinitis is caused by photochemical damage to the retina by light with a wavelength towards the lower end of the visible spectrum [10]. The maximum sensitivity of the retina to such damage is around 445 nm in phakic eyes; at 440 nm the damage threshold for a retinal lesion is approximately 22 J/cm^2^ [21]. Visible light with peak wavelength at 440 nm was measured at the retina during flap cutting with a 345 nm femtosecond laser in porcine eyes [6]. This light is emitted from the ocular media following absorption of UV light. The “blue light” represents the most hazardous component of the visible spectrum with the greatest potential for phototoxicity [34]. Le Harzic et al. estimated that approximately 2% of the energy dose of the UV laser light reaches the retina as visible light during flap processing [6]. At a radiant exposure of 18 J/cm^2^ (“maximum energy” as used in this study), the retinal damage threshold is not surpassed and no deleterious effect on the retina should be expected. Our data confirm this hypothesis.

Long-term exposure of Dutch Belted rabbits to visible light resulted in ERG changes and histopathologic evidence of RPE and photoreceptor inner and outer segment damage [35]. In a more recent study in rabbits, Wang et al. also found a reduction of ERG amplitudes and progressive photoreceptor damage up to three months following UVA crosslinking of the sclera [36]. Since in our experiments no effects on ERG measurements, no alteration of retinal morphology and no reduction of retinal cell numbers was observed, we are confident that retinal cell death due to photochemical damage [22] did not occur. However, it should be considered that the retinas of our young experimental animals were healthy, and vulnerability to photochemical damage, particularly in species that have a macula lutea, may be increased by age [34] and pre-existing retinal pathology [22].

Dutch Belted rabbits vary considerably in fundus pigmentation both between animals and within the same fundus [35]. Energy absorption by melanin pigment of the RPE is critical for the creation of photothermal retinal damage [34], but this mechanism is not expected to play a predominant role during the use of the femtosecond UV laser [37]. Indeed, no evidence for photothermal damage could be detected in our histopathological analysis. Instead, it has been suggested that melanin may have a protective role against photochemical effects due to antioxidant properties [22]. However, Noell et al. found no difference between pigmented and non-pigmented rats with regards to ERG changes caused by monochromatic light at 435 nm [38]. Also, Lawwill found no difference between strongly pigmented areas and less strongly pigmented retinal areas with respect to light induced damage in his study on Dutch Belted rabbits [35]. Accordingly, in our series, no difference between individual animals could be discerned.

In summary, no damage to lens and retina could be detected at the maximum levels of radiant exposure applied during this study. Photomechanical disruption of corneal tissue is achieved at much lower energy levels, so that in practice the maximum dose would not be reached. Our data suggest that the refractive 345 nm femtosecond laser is safe with respect to intraocular radiation hazard.

## Supporting Information

S1 Checklist NC3Rs ARRIVE Guidelines Checklist (PDF)

S1 Fig Representative histological sections of iris stroma. No alterations were found in the iris of treated (A) or untreated (B) eyes. S: iris stroma; asterisk: sphincter pupillae muscle; arrows: pigmented iris epithelium lined by dilator pupillae muscle. H&E stain.
(JPG)
