# Supplementary figures and images for: Evaluation of a 345 nm Femtosecond Laser for Corneal Surgery with Respect to Intraocular Radiation Hazard
Source: PLoS One. 2015 Sep 11;10(9):e0137638. doi: 10.1371/journal.pone.0137638 (PMC4567343; doi:10.1371/journal.pone.0137638)

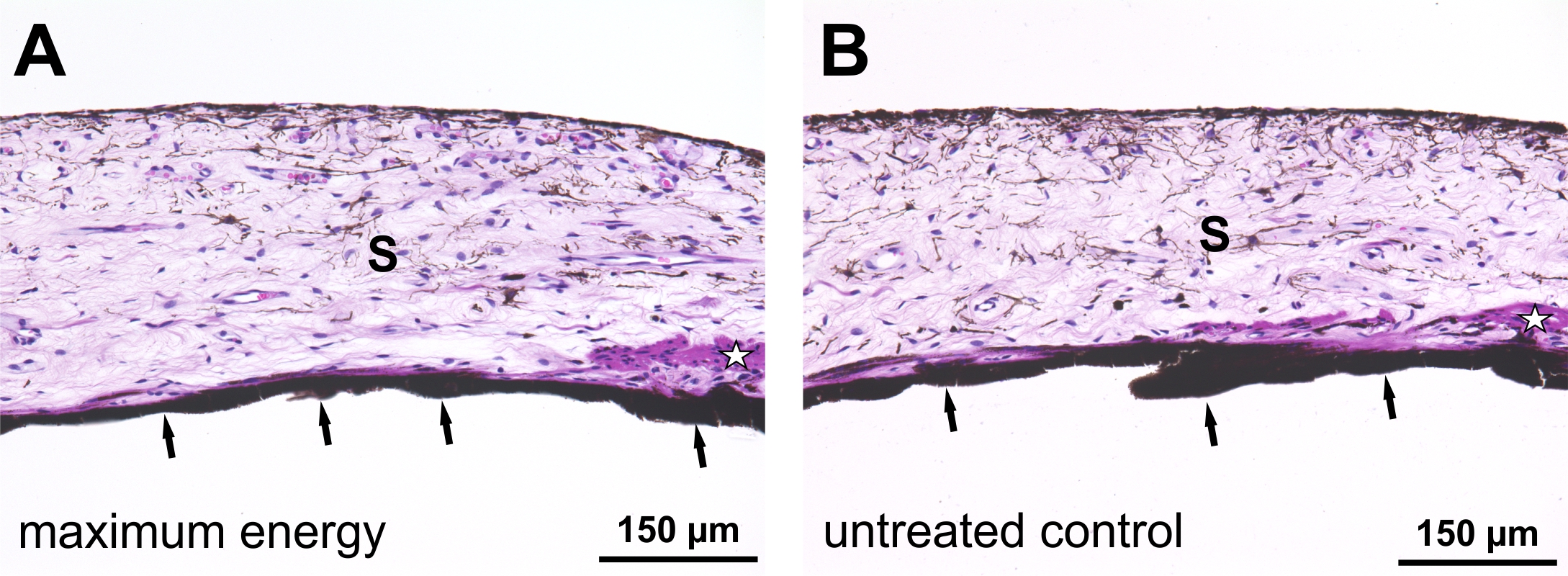

Supplement: S1 Fig — No alterations were found in the iris of treated (A) or untreated (B) eyes. S: iris stroma; asterisk: sphincter pupillae muscle; arrows: pigmented iris epithelium lined by dilator pupillae muscle. H&E stain. (JPG) [file pone.0137638.s002.jpg]
